# Heterogeneity in predictive power of early childhood nutritional indicators for mid-childhood outcomes: Evidence from Vietnam

**DOI:** 10.1016/j.ehb.2017.02.002

**Published:** 2017-08

**Authors:** Le Thuc Duc, Jere R. Behrman

**Affiliations:** a Centre for Analysis and Forecasting, Vietnam Academy of Social Sciences, No.1, Lieu Giai Road, Hanoi, Viet Nam; b Economics and Sociology Departments and Population Studies Center, University of Pennsylvania, McNeil 160, 3718 Locust Walk, Philadelphia, PA 19104-6297, USA

**Keywords:** Birth weight, Weight gain, Height growth, Child anthropometry, Education, Vietnam

## Abstract

We utilize longitudinal data on nearly 1800 children in Vietnam to study the predictive power of alternative measures of early childhood undernutrition for outcomes at age eight years: weight-for-age (WAZ8), height-for-age (HAZ8), and education (reading, math and receptive vocabulary). We apply two-stage procedures to derive unpredicted weight gain and height growth in the first year of life. Our estimates show that a standard deviation (SD) increase in birth weight is associated with an increase of 0.14 (standard error [SE]: 0.03) in WAZ8 and 0.12 (SE: 0.02) in HAZ8. These are significantly lower than the corresponding figures for a SD increase in unpredicted weight gain: 0.51 (SE: 0.02) and 0.33 (SE: 0.02).

The heterogeneity of the predictive power of early childhood nutrition indicators for mid-childhood outcomes reflects both life-cycle considerations (prenatal versus postnatal) and the choice of anthropometric measure (height versus weight). Even though all the nutritional indicators that involve postnatal nutritional status are important predictors for all the mid-childhood outcomes, there are some important differences between the indicators on weight and height. The magnitude of associations with the outcomes is one aspect of the heterogeneity. More importantly there is a component of height-for-age z-score (at age 12 months) that adds predictive power for all the mid-childhood outcomes beyond that of birth weight and weight gain in the first year of life.

## Highlights

• Birth weight, weight gain and height growth predict height and weight at 8 years.
• Weight gain and height growth also predict educational outcomes at 8 years.
• The predictive power of the unpredicted weight gain exceeds that of birth weight.
• Height growth adds predictive power beyond that of birth weight and weight gain.
• Height growth predicts receptive vocabulary, unlike birth weight and weight gain.

## 1 Introduction

Studies on the importance of early-life anthropometry for later human capital development recently have become prominent. Behrman and Rosenzweig (2004), Black et al. (2007), Victora et al. (2008), Rosenzweig and Zhang (2013), and Figlio et al. (2014) find birth weight to have significant associations with long-run adult health, education and earnings. Gupta et al. (2011) and Krishna et al. (2016) are the most relevant previous studies for the birth weight-related contents in our study.^1^ Gupta et al. (2011) use data from the Danish Longitudinal Survey of Children (DALSC), which followed children born in 1995 with surveys in 1996, 1999, 2003 and 2007. For mid-childhood outcomes, their findings imply that the associations of low birth weight (2.5 kg or lower) with weight and height are statistically significant, but not the associations with behavioral outcomes. The data used in Krishna et al. (2016) are from Young Lives, which is described in the next section. They find that prenatal conditions, reflected in birth weight, are more strongly associated with height trajectories than postnatal factors; they do not consider weight and educational outcomes.

A number of child health and nutrition researchers have focused on the concept of a critical window for investing in early childhood nutrition during the first 1000 days after conception (Martorell et al., 1994, Victora et al., 2008, Victora et al., 2010, Prentice et al., 2013, Lundeen et al., 2014). Stunting at age 2–3 years, which is indicated by deficits of two standard deviations or more below the median height-for-age for a well-nourished reference population, has become an important policy concern (Engle et al., 2007, Engle et al., 2011, Grantham-McGregor et al., 2007, UNICEF, 2013, Richter et al., 2016).

Inadequate prenatal nutrition results in low birth weight and inadequate postnatal nutrition results in low weight and height gains in early childhood. There have been few studies that focus on the impacts of infant weight gain and height growth. Even fewer studies investigate the relative importance for predicting later development of weight gain versus height growth. Huang et al. (2013) reviews the limited literature on the predictive power of infant weight gain and height growth and reports substantial variety in the findings. Li et al. (2004) find that height growth during the postnatal period (birth to age 2 years) is the only variable predictive of Guatemalan women’s educational achievement and weight gain is not statistically significant. Corbett et al. (2007) find in UK data that “postnatal weight gain in the first 2 years of life is at most weakly related to cognitive and education attainment at age 10. In contrast, birth weight is clearly associated with cognitive and educational attainment at age 10…” (2007: 62). There is, however, an issue that may affect Corbett et al’s results: factors such as birth order, maternal education and family environment are not included in their analysis. More recently, Huang et al. (2013) evaluated the relative associations of birth weight and postnatal growth (weight gain, height growth, or head circumference growth) with cognition and behavioral development in over 8000 Chinese children. They find that, for full-term children, both birth weight and postnatal growth are associated with child’s IQ at age 4–7 years, but the sizes of the associations are small.

While Huang et al. (2013) used up to seven years for their postnatal period, we consider the postnatal period of the first year, which is within the usually-emphasized critical window. We study the nexus of the early childhood undernutrition with anthropometric and educational outcomes in mid childhood. There are many studies that use height-for-age z-scores to predict longer-run outcomes and many that use birth weight (e.g., Crookston et al., 2013, Victora et al., 2008). While height-for-age is often interpreted to measure the nutritional status over the whole period from conception to measurement, weight-for-age is expected to have an advantage in capturing the effect of recent health shocks before the survey, such as diarrhea in the few months before the survey. In the same vein, weight gain after birth might be relevant in addition to birth weight and weight-for-age. The timing of the impact is important because of the debate on the “critical period” during which brain development is most sensitive to poor nutrition. Dobbing (1976), for example, argues that the period from birth to six months is the most critical. For other authors, such as Doyle et al. (2009), the prenatal period is more important.

To separate the pre- versus postnatal influences of nutrition status, we apply two-stage procedures in which we derive indicators of unpredicted nutritional changes. We define the unpredicted weight gain (height growth) in the first year of life as the component of weight-for-age (height-for-age) z-score at age 12 months that is unpredicted based on what is known at birth, including birth weight. Further, we define the *conditionally* unpredicted height-for-age as the component of the unpredicted height growth that is uncorrelated with weight-for-age at age one year. The conditionally unpredicted height-for-age is useful for comparison of predictive powers of the indicators on height growth versus that of weight gain in the first year of life.

The sample of children we work with in this study differs from those in the aforementioned studies on birth weight in a number of dimensions. Our data contain no twins, so the distribution of birth weight differs from that in the papers that use data on twins since the distribution of birth weights for twins is to the left of the distribution of birth weights for singletons.^2^ In part because our data do not have twins, less than five percent of children in our sample have birth weight under 2.5 kg (the standard cutoff for low birth weight), even though we use a semi-purposeful pro-poor sample from Vietnam. Also the age patterns of undernourishment are very different than reported for other contexts in previous studies. In the first decade of the 21st century, for Vietnamese under-5 years old moderate and severe stunting rates were as high as those for West and Central Africa, while the Vietnamese percentage of low birth weights was the same as for high-income countries (UNICEF, 2009).

We examine different combinations of variables for birth weight, weight-for-age, height-for-age, unpredicted weight gain (height growth) and conditionally unpredicted weight-for-age (height-for-age), together with a set of controls, to estimate their associations with mid-childhood outcomes. We find that a standard deviation higher unpredicted weight gain (height growth) in the first year of life generally is more associated with positive outcomes in mid-childhood than are the adverse outcomes associated with a standard deviation lower birth weight. This suggests that for most of the children, the adverse outcomes related to being born low weight may be partially or totally avoided by weight gain (height growth) in the first postnatal year. In addition to the question of prenatal versus postnatal timing, we also investigate another aspect of heterogeneity of the relationships of the early childhood nutrition indicators and mid-childhood outcomes: the difference between height and weight. We find that height-for-age z-score at age one year contains a component that adds explanatory power for the variation of all the mid-childhood outcomes, beyond that of birth weight and weight gain in the first year of life.

## 2 Methods

### 2.1 Young lives data for Vietnam

The sample is part of Young Lives, which is an international comparative study of child poverty. Since 2002, Young Lives has been following ∼12,000 children in Ethiopia, India, Peru and Vietnam. The Young Lives sample consists of two cohorts: ∼8000 children born in 2001–2 and ∼4000 children born in 1994–5. The Vietnamese Younger Cohort sample, which we use in this study, consists of 20 commune-based clusters in Northern Uplands, Red River Delta, Central Coast, and Mekong Delta. The subsamples for each of these regions contain four rural clusters, except that for the Central Coast, which consists of four urban clusters in the city of Da Nang and four rural ones in the province of Phu Yen. The children in this study were on average 11.6 months old in Round 1, with the youngest being 5 months old and the oldest being 18 months old. Consistent with the design of the Young Lives study, we limit consideration to children in the age range from 6 to 17.9 months and therefore exclude 21 observations.^3^ Furthermore, following the same method as in Lundeen et al. (2014) and Schott et al. (2013), we exclude 45 observations with anthropometric measures in Rounds 1–3 either too low or too high or with unusually large changes (in height or weight) from one survey round to another.^4^ We also drop a further 65 observations because of missing data on either weight or height in Rounds 1–3. There are missing values on other variables discussed in the following section.

### 2.2 Variables

As presented in Table 1, the characteristics of children include: sex, birth order, birth weight, year of birth, weight at one year, and length at one year. To allow nonlinearity in the effect of birth weight at the high end, we use a dummy variable that equals 1 for birth weight >3.5 kg.^5^ The children under study are divided into two groups by birth year. A dummy variable is defined for the children born in 2002. By the Education Law of Vietnam, the children in this group started school one year after the ones born in 2001, regardless of birth month. The Law was not strictly followed, however.^6^

**Table 1:** Descriptive statistics of variables.

Variables	Mean	Std. Dev.
Birth weight (kg)	3.10	0.45
Birth weight greater than 3.5 kg	0.12	0.32
Missing birth weight	0.11	0.32
If boy	0.51	0.50
Born in 2002	0.19	0.40
Height-for-age z score in Round 1	−1.06	1.15
Weight-for-age z score in Round 1	−0.93	1.02
Height-for-age z score at age 8 years (HAZ8)	−1.06	1.01
Weight-for-age z score at age 8 years (WAZ8)	−1.12	1.26
EGRA (proportion correct)	0.50	0.12
Math (proportion correct)	0.68	0.20
Receptive Vocabulary (proportion correct)	0.47	0.14
If mother’s first child	0.46	0.50
Mother’s height (cm)	152.4	5.65
Mother’s BMI	21.06	2.68
Mother is ethnic minority	0.12	0.32
Mother completed lower secondary school	0.36	0.48
Father completed lower secondary school	0.45	0.50
Wealth index in Round 1	0.44	0.21
Urban	0.20	0.40
Mountainous	0.23	0.42
Red River Delta	0.21	0.41
Mekong Coastal^a^	0.10	0.30
Mekong Delta	0.20	0.40

a Half of Mekong Delta, with significance differences in parental education and services.

Data on birth weight were recorded in birth documents by health clinic staff at the time of birth. Other anthropometric data were collected in the Young Lives surveys. Birth weight has missing observations for 11% of the children. We impute values for these missing observations on the basis of an OLS regression for the 89% of the sample that has birth weight data and the main caregiver’s recollection (at age 1 year) of the child’s size at birth and other relevant factors (Appendix A). We normalize birth weight in standard deviation (SD) units for our estimates to facilitate comparisons of coefficient magnitudes across the nutritional indicators by having them all in SD units. Supine length (Round 1) and standing height (Rounds 2&3) were measured with length/height boards using standardized WHO methodology (WHO, 2008) and measurements precise to 1 mm. Weights were measured precise to a tenth of a kilogram (kg). The height (weight) measurements were converted into height-for-age z scores (HAZ) and weight-for-age z scores (WAZ) using WHO standards (WHO, 2006a, WHO, 2006b, de Onis et al., 2007) based on well-nourished populations. As mentioned earlier, child ages varied between 6 and 17.9 months in Round 1 in our sample. In the analysis that follows, it is desirable to convert these Round 1 anthropometric measures to the same age in months because on average in this and in most undernourished populations HAZ declines significantly over this age range (e.g., Victora et al., 2010). Following the method used in previous studies (e.g., Stein et al., 2010, Crookston et al., 2013, Schott et al., 2013) we *project* the value of HAZ for the children either older or younger than 12 months as follows. The average HAZ for age *i* (in months) is defined as the mean of HAZ for all the children age *i* − 1, *i* and *i* + 1 months.^7^ The projected value of HAZ at age 12 months is the HAZ in Round 1 for each child minus the age-group average HAZ for the actual age of the child in Round 1 plus the average HAZ for children 12 months of age in Round 1. That is, each child is presumed to be the same number of HAZ units above or below the average HAZ curve for all children at age 12 months as the child was at the age actually measured.^8^ The projected value of HAZ at age 12 months is denoted by HAZ*1*. We also apply this method, using WAZ in Round 1 to calculate the projected WAZ at age 12 months, denoted by WAZ*1*. All the outcomes are assessed at age eight years. The anthropometric outcomes are height-for-age (HAZ*8*) and weight-for-age (WAZ*8*). The educational outcomes are the test scores in Early Grade Reading Assessment (EGRA), math and Peabody Picture Vocabulary Test (PPVT) the children took at age eight years. The PPVT assesses the receptive vocabulary outcome. Cueto and Leon (2012) analyze the psychometric characteristics of the math test and the PPVT.

Household characteristics include: mother’s height, mother’s weight, mother’s ethnicity, mother’s schooling attainment, father’s schooling attainment, and a wealth index in Round 1. The wealth index is the simple mean of three components: (1) housing quality, which is the scaled (0–1) mean rooms per person as a continuous variable and dummies on floor, roof and walls^9^; (2) the value of consumer durables, which is the scaled (0–1) sum of nine dummies for basic consumer durables^10^; and (3) the value of health-related infrastructural services, which is the simple mean of drinking water, electricity, sanitation facilities, and fuel, all of which are 0–1 variables.^11^

Regional characteristics are represented by dummy variables for Urban, Red River Delta, Mekong Delta, and Mountainous. We apply the official list by Committee for Ethnic Minorities of Vietnam to define the Mountainous category.^12^ Mountainous areas in Vietnam are considered less developed than other areas. Among the mountainous clusters, however, we find one with average birth weight above that of whole sample. Looking into the details, we find high concentration of Tay and Nung ethnic groups in this cluster. Together with the ethnic majority of Kinh, these ethnic groups count for 84% of observations in this cluster. These ethnic groups do relatively well in economic development, and in the decade under research, ethnic minorities groups Tay and Nung were more prosperous than the other ethnic minority groups in the Young Lives sample. For these reasons, we add a dummy variable for this cluster. Similarly, another dummy variable is included for the coastal part of the Mekong region because these sites are distinguished considerably from the other part of the Mekong region. The gaps between the coastal and the inlands of Mekong are most significant in parental schooling and services in general.^13^

Our analysis involves many variables from multiple rounds of longitudinal data. So potentially there might be a problem of missing values beyond that discussed above with regard to birth weight. We find, however, that there is no concentration of missing values for any particular variable or for any group of children. The final panel consists of 1758 observations, or 89% of the children aged 6–17.9 months from the initial sample.

### 2.3 Model specifications

In addition to the variables in Table 1, we work with some components of the postnatal nutritional indicators. The use of these components involves two-stage procedures that are defined below. For the definition of unpredicted components, we would ideally use the control factors at birth, but data on the household and community characteristics were collected for the first time in Round 1, when the children were approximately one year old. Therefore we assume that the control factors are stable from birth to Round 1.^14^ With that assumption, we define the components of WAZ1 and HAZ1 that cannot be predicted based on all the information available at birth. The unpredicted weight gain during the first year of life is defined as the residual *y*_1_ in the following ordinary least squares (OLS) regression:

(1a) $$WAZ1=\pi_{0}+\pi_{1}y_{0}+X\text{'}\Pi +y_{1}$$

where *y*_0_ denotes birth weight, *X* is the set of non-child nutrition control factors, and Π is a vector of coefficients for the controls. Similarly, the unpredicted height growth during the first year of life is defined as the residual *y*_2_ in the following regression:

(1b) $$HAZ1=\mu_{0}+\mu_{1}y_{0}+X\text{'}M+y_{2}$$

with M being another vector of coefficients. For these equations the right-side child, household and community characteristics refer to a specific age (baseline when the child was one year old) and therefore, to limit clutter, age indexes are not indicated. Furthermore, we define the conditionally unpredicted weight-for-age at 12 months as *y*_3_, the residual in the following regression:

(2a) $$WAZ1={\bar{\pi }}_{0}+{\bar{\pi }}_{1}y_{0}+{\bar{\pi }}_{2}HAZ1+X^{\prime }\bar{\text{Π}}+y_{3}$$

Similarly, the conditionally unpredicted height-for-age at 12 months is *y*_4_, the residual in the following regression:

(2b) $$HAZ1={\bar{\mu }}_{0}+{\bar{\mu }}_{1}y_{0}+{\bar{\mu }}_{2}WAZ1+X^{\prime }\bar{\text{Μ}}+y_{4}$$

Even though the unpredicted terms *y*_1_ and *y*_2_ correlate strongly with the corresponding z-scores, they are not perfectly correlated so there is some leeway for having differential predictive power. The correlation between WAZ1 and *y*_1_ is 0.90, and that for HAZ1 and *y*_2_ is 0.86. The correlation between *y*_1_ and *y*_2_ is weaker than that between WAZ1 and HAZ1 (0.69 against 0.74). The residual *y*_3_ is orthogonal to birth weight, height-for-age and all the regressors in *X*, and is therefore, orthogonal to the unpredicted height growth *y*_2_ as well. Symmetrically, the residual *y*_4_ is orthogonal to birth weight, the unpredicted weight gain *y*_1_ and all the controls.

The associations of the nutritional variables with the outcomes are the estimated coefficients in the following equation:

(3) $$C=\alpha +\beta_{0}y_{0}+\beta_{1}z_{1}+\beta_{2}z_{2}+X\text{'}B+u$$

where *C* denotes one of the anthropometric (HAZ8 or WAZ8), educational (EGRA, math or PPVT) outcomes at age eight years; *y*_0_ is birth weight, a measure of pre-natal nutritional status; *z*_1_ and *z*_2_ are two measures of postnatal nutritional status that are defined below for different models. The set of control factors *X* is the same as in Eqs. (1a), (1b) (2a), and (2b). Finally, *u* is the error term.

We consider two groups of models corresponding to the following groupings of variables *y*_0_, *z*_1_ and *z*_2_. In both groups there is a set of models (Models 1–2 in Group I and Models 3–4 in Group II) that include one relation with a weight-related indicator as the dependent variables for Eqs. (1a) and (2a) (indicated by W) and one relation with a height-related indicator as the dependent variables for Eqs. (1b) and (2b) (indicated by H). Group I consists of models that include zero or one of the early childhood nutritional variables:

▓▓*Model C*: controls only, no variable on child nutritional status.^15^

▓▓*Model 0*: birth weight *y*_0_ is the only nutritional variable, e.g. *β*_1_ = *β*_2_ = 0.

▓▓*Model 1W*: WAZ1 represents *z*_1_, *β*_0_ = *β*_2_ = 0;

▓▓*Model 1H*: HAZ1 represents *z*_2_, *β*_0_ = *β*_1_ = 0;

▓▓*Model 2W*: *y*_1_ (Eq. (1a)) represents *z*_1_, *β*_0_ = *β*_2_ = 0;

▓▓*Model 2H*: *y*_2_ (Eq. (1b)) represents *z*_2_, *β*_0_ = *β*_1_ = 0.

In Group II, all models contain birth weight and one or two additional nutritional variables:

▓▓*Model 3W*: birth weight *y*_0_ and *y*_1_ represents *z*_1_, *β*_2_ = 0;

▓▓*Model 3H*: birth weight *y*_0_ and *y*_2_ represents *z*_2_, *β*_1_ = 0;

▓▓*Model 4HW*: birth weight *y*_0_, *y*_2_ presents *z*_1_ and *y*_3_ (Eq. (2a)) represents *z*_2_.

▓▓*Model 4WH*: birth weight *y*_0_, *y*_1_ presents *z*_1_, and *y*_4_ (Eq. (2b)) represents z_2_.

By estimation of Model C, we will find the explanatory power of the controls, and therefore can assess how much the nutritional variables added in the other models increase the predictive power in the estimation of Eq. (3). Models in Group I are used for estimation of the associations for each individual early-life nutritional variables with the outcomes. Models in Group II contain two or three variables, of which at least one captures postnatal nutrition. These specifications are to demonstrate the relative importance of postnatal nutritional indicators. The purpose of Model 4HW, for instance, is to investigate if weight-for-age, which contains *y*_3_ as a component, adds to the predictive power beyond that of the combination of birth weight and the unpredicted height growth. On the other hand, Model 4WH permits investigation of whether the height-for-age, which contains *y*_4_, has predictive power beyond that of birth weight and the unpredicted weight gain in the first year of life.

## 3 Estimates

Ordinary Least Squares (OLS) regressions are used for all the estimations. We relax the requirement that the observations be independent, allowing for the possibility that community factors cause correlations within communes that results in heteroskedasticity. To increase the likelihood that the standard errors are “robust” to heteroscedasticity, we apply an option aiming at robust estimators. For the estimates in Tables Table 2, Table 3, Table 4 and A2 (in Appendix B) , the Stata option used in the OLS estimations is vce(cluster community), with community being the commune id.

**Table 2:** Estimates for coefficients of the controls for Model C.

	WAZ8	HAZ8	EGRA	Math	PPVT
If boy	0.040	−0.083**	−0.141***	−0.017	0.025
	(0.060)	(0.040)	(0.051)	(0.028)	(0.039)
If mother’s first child	0.329***	0.133**	0.112*	0.065	0.072
	(0.081)	(0.051)	(0.055)	(0.041)	(0.051)
Born in 2002	0.028	0.007	−0.303***	−0.868***	−0.405***
	(0.067)	(0.054)	(0.056)	(0.068)	(0.078)
Mother's height (norm.)	0.211***	0.292***	0.041*	0.032*	0.019
	(0.026)	(0.025)	(0.022)	(0.017)	(0.022)
Mother’s BMI	0.102***	0.026***	−0.007	−0.007	0.003
	(0.010)	(0.009)	(0.010)	(0.008)	(0.008)
Mother ethnic minority	−0.057	−0.280***	−0.500***	−0.541***	−0.400***
	(0.079)	(0.076)	(0.165)	(0.173)	(0.112)
Mother completed Lower Secondary Education (LSE)	0.236***	0.162***	0.176**	0.180***	0.322***
	(0.072)	(0.049)	(0.066)	(0.051)	(0.071)
Father completed LSE	0.079	0.069	0.120**	0.154***	0.103**
	(0.052)	(0.044)	(0.055)	(0.046)	(0.048)
Wealth index R1	0.284***	0.160***	0.146**	0.211***	0.233***
	(0.066)	(0.050)	(0.060)	(0.061)	(0.068)
Urban	0.666**	0.449***	0.489**	0.163	0.311
	(0.269)	(0.148)	(0.211)	(0.222)	(0.225)

*Notes*: The effects of region and specific sites are not displayed; the test scores in EGRA, Math and PPVT are normalized by the sample standard deviations; Number of observations: 1758; the standard errors (in parentheses, underneath the coefficients) are robust to heteroscedasticity.

* p < 0.1.

** p < 0.05.

*** p < 0.01.

**Table 3:** Estimates of coefficients of nutritional variables for Models in Group I.

Model	Variables other than controls	WAZ8	HAZ8	EGRA	Math	PPVT
C	R-squared ($R_{C}^{2}$)	0.314	0.282	0.175	0.345	0.281

0	Norm. birth weight (y_0_)	0.153***	0.134***	−0.005	0.009	−0.017
		(0.031)	(0.027)	(0.032)	(0.036)	(0.025)
	R^2^- $R_{C}^{2}$	0.014	0.011	0.001	0.004	0.002

1W	Weight-for-age at 1 year	0.561***	0.376***	0.119***	0.097***	0.024
		(0.022)	(0.020)	(0.022)	(0.022)	(0.022)
	R^2^	0.489	0.402	0.188	0.357	0.283

1H	Height-for-age at 1 year	0.436***	0.478***	0.106***	0.100***	0.041**
		(0.027)	(0.021)	(0.021)	(0.017)	(0.018)
	R^2^	0.436	0.500	0.187	0.359	0.284

2W	Unpredicted weight gain (y_1_)	0.507***	0.336***	0.113***	0.090***	0.025
		(0.021)	(0.018)	(0.021)	(0.019)	(0.020)
	R^2^	0.482	0.394	0.189	0.357	0.283

2H	Unpredicted height growth (y_2_)	0.418***	0.461***	0.106***	0.099***	0.043**
		(0.026)	(0.022)	(0.022)	(0.017)	(0.017)
	R^2^	0.430	0.493	0.188	0.359	0.285

*Notes*: The variables on early childhood nutrition and on test scores in EGRA, Math and PPVT are normalized by the sample standard deviations; Number of observations: 1758; the standard errors (in parentheses, underneath the coefficients) are robust to heteroscedasticity.

^*^p < 0.1.

** p < 0.05.

*** p < 0.01.

**Table 4:** Estimates of coefficients of nutritional variables for Models in Group II.

Model	Variables other than controls	WAZ8	HAZ8	EGRA	Math	PPVT
3W	Birth weight (y_0_)	0.141***	0.126***	−0.007	0.006	−0.018
		(0.025)	(0.022)	(0.033)	(0.035)	(0.025)
	Unpredicted weight gain (y_1_)	0.505***	0.334***	0.113***	0.090***	0.025
		(0.020)	(0.018)	(0.022)	(0.019)	(0.020)
	R^2^	0.489	0.403	0.189	0.357	0.284

3H	Birth weight (y_0_)	0.140***	0.120***	−0.008	0.005	−0.019
		(0.027)	(0.022)	(0.032)	(0.035)	(0.025)
	Unpredicted height growth (y_2_)	0.415***	0.459***	0.107***	0.099***	0.043**
		(0.026)	(0.022)	(0.022)	(0.017)	(0.017)
	R^2^	0.436	0.500	0.188	0.359	0.285

4HW	Birth weight (y_0_)	0.139***	0.119***	−0.008	0.005	−0.019
		(0.025)	(0.022)	(0.033)	(0.035)	(0.025)
	Unpredicted height growth (y_2_)	0.415***	0.459***	0.107***	0.099***	0.043**
		(0.022)	(0.022)	(0.022)	(0.017)	(0.017)
	Conditional unpredicted WAZ1 (y_3_)	0.303***	0.026	0.055***	0.030*	−0.007
		(0.023)	(0.023)	(0.020)	(0.017)	(0.018)
	R^2^	0.494	0.501	0.191	0.360	0.285

4WH	Birth weight (y_0_)	0.139***	0.119***	−0.008	0.005	−0.019
		(0.025)	(0.022)	(0.033)	(0.035)	(0.025)
	Unpredicted weight gain (y_1_)	0.506***	0.335***	0.113***	0.090***	0.025
		(0.020)	(0.015)	(0.021)	(0.019)	(0.020)
	Conditional unpredicted HAZ1 (y_4_)	0.093***	0.316***	0.040*	0.051***	0.036**
		(0.025)	(0.027)	(0.020)	(0.015)	(0.015)
	R^2^	0.494	0.501	0.191	0.360	0.285

*Notes*: The variables on early childhood nutrition and on test scores in EGRA, Math and PPVT are normalized by the sample standard deviations; Number of observations: 1758; the standard errors (in parentheses, underneath the coefficients) are robust to heteroscedasticity.

* p < 0.1.

** p < 0.05.

*** p < 0.01.

### 3.1 Estimates for controls

As described above, there is no nutritional indicators among the controls for Model C. The estimates for Model C are presented in Table 2. It can be seen that the gender factor (being a boy) is negatively significantly associated with height-for-age at eight years of age. This association means that the gap in HAZ8 between eight-year boys in Vietnam and the boys of the same age in the WHO sample is less favorable than the gap between eight-year girls in Vietnam and the same-aged girls in the WHO sample. The estimate for gender also implies that boys perform poorer than girls in EGRA. The gender gaps are not statistically significant for the other outcomes. Being first-born is positively significantly associated with anthropometric outcomes and moderately significantly associated with EGRA. Birth year is an important predictor for the educational outcomes, but not for anthropometric outcomes. The omitted category for the year of birth consists of children born in 2001, so the results imply that older children perform better than younger ones. Schooling might have been a part of reason. In fact, in the academic year 2009–10, 92% of the children born in 2001 were in grades 3–4, while only 30% of children born in 2002 were in grade 3 or higher.

Mother’s anthropometrics are significant for child anthropometric outcomes at age eight years. With respect to the educational outcomes, there is slight difference between the two anthropometric indicators for mothers. Mother’s height is moderately associated with the EGRA and math tests. The children of ethnic minority mothers perform significantly less well than those of ethnic majority mothers for all the outcomes, except weight. Socioeconomic status (parental schooling and wealth index) is significant for all the educational outcomes. For the child anthropometric outcomes, the associations of mother’s schooling are statistically significant, but those of father’s schooling are not. Finally, residing in the urban sector has statistically significant associations with anthropometric outcomes and reading.

### 3.2 Basic estimates for early childhood nutrition indicators

As discussed in Section 2, 11% of the children have missing data on birth weight, and for these observations, the imputed values are used in the same way as the actual data for the regressions in Table 2. For the estimates in Table 3, Table 4, in addition to the controls used in Model C, a dichotomous variable for “Birth weight imputed (1/0)” is included, as well as the dummy variable on “Birth weight greater than 3.5 kg”. To ease any concern about possible bias related to imputation of data on birth weight, we also run regressions on the reduced sample with only observations that include birth weight data, excluding the imputed values. The estimates are presented in Table A2 in the Appendix B.

Table 3 presents OLS multivariate regression estimates for Group I. The R-squareds for Model C imply that between 18% and 35% of the variations in the outcomes are predicted by the variation in the controls. The differences $R^{2}-R_{C}^{2}$ for the Model 0 show how much predictive power the nutritional indicator *y*_0_ (together with the aforementioned dummy variables) add beyond that of the controls. In case of Model 0, it makes very little difference in R-squared (one percent for the regressions for the anthropometries and almost none for the other outcomes). The estimates for Model 0 imply that birth weight is strongly associated with both anthropometric outcomes at age eight years. With the educational outcomes, however, at any reasonable statistical significance level, we cannot reject the hypothesis of no association with birth weight.

For the remaining models, we present *R*^2^, rather than the differences, which are significant at least for the anthropometric outcomes, unlike that for Model 0. The indicator of weight-for-age at age one year WAZ1 in Model 1W is strongly associated with all the outcomes, except PPVT. The goodness of fit increased from that for Model 0, and the change is most significant for the WAZ8 outcome (0.17, or more than 50%, above that for Model C). The estimates for the nutritional indicator in Model 1W are very similar to the corresponding ones in Model 2W. Weight-for-age z-score at age one year and the unpredicted weight gain in the first year of life are statistically significantly associated with reading and math outcomes, unlike birth weight.

The estimates imply that for the anthropometric outcomes, the explanatory power of WAZ1, which includes prenatal nutrition status plus postnatal weight gain, is slightly greater than that of *y*_1_. For the non-anthropometric outcomes, however, R-squares in Model 1W are almost identical to the corresponding R-squares in Model 2W, which is further evidence that variation in birth weight has little predicative power for the educational outcomes. For Model 1H, in contrast to Models 0 and 1W, there is a statistically significant association between the nutritional indicator, in this case HAZ1, and PPVT. The estimates for Model 1H are very similar to those for Model 2H. The estimate for HAZ8 in Model 1H is consistent with previous literature, such as Krishna et al. (2016).^16^

The estimates for Models 3W and 3H in Table 4 are broadly consistent with the parallel estimates in Models 2W and 2H. However, the inclusion of birth weight together with the unpredicted weight gain (height growth) allows comparisons of the predictive power of these variables. The estimates for Model 3W suggest that a one SD increase in the unpredicted weight gain in the first year is associated with variation in the anthropometric outcomes more than double of those for a one SD increase in birth weight. For the EGRA and math scores, the estimated associations of a SD change for the unpredicted weight gain are clearly more significant than the corresponding estimates for a SD change of birth weight. Neither *y*_0_ nor *y*_1_ is statistically significantly associated with PPVT.

As for Model 2H, unpredicted height growth [in Model 3H] is associated significantly with PPVT – that is not the case for the unpredicted weight gain in Models 2W and 3W. Estimates for Model 3H imply that a child born with one SD of birth weight under the mean but with one SD of unpredicted height growth in the first year of life is expected to have better outcomes in mid-childhood than a child born with average birth weight who grew normally (as predicted) in the first year of life.

The estimates for Model 4HW suggest that with the presence of the unpredicted height growth (*y*_2_) among the regressors, the predictive power of the conditional unpredicted weight-for-age *y*_3_ is not statistically significant for the height and PPVT outcomes, and for the math outcome it is not statistically significant at five percent. Related to these estimates, the corresponding results in Table A2 are not statistically significant for all the outcomes in height, math and PPVT.

On the other hand, the conditional unpredicted height-for-age *y*_4_ in Model 4WH is statistically significantly associated with all the outcomes. In fact, we can see that *y*_4_ contributes to prediction of variations in anthropometric outcomes, math and PPVT, all at the level of five percent significance. At the ten percent significance level, one can reject the hypothesis that *y*_4_ has no association with EGRA. In Table A2, however, the estimate for *y*_4_ is statistically significant at five percent for all the outcomes. By definition, the conditionally unpredicted height-for-age *y*_4_ is orthogonal to birth weight *y*_0_, the unpredicted weight gain *y*_1_ and all the control factors. That means the variations of the outcomes that are predicted by *y*_4_ are beyond those predicted by *y*_0_, *y*_1_ and all the control factors. Finally, it can be seen in Tables 3, 4 and A2 that there are slight differences between the two sets of estimates concerning the magnitudes but not the statistical significance, except for estimates for *y*_3_ and for *y*_4_ in Model 4WH and 4HW, as discussed in the preceding paragraph. There are no such inconsistencies with respect to the associations of the indicators with birth weight or any of the z-scores, or unpredicted terms *y*_1_ and *y*_2_. Overall, the results in Table A2 are consistent with the basic findings in Table 3, Table 4.

## 4 Discussion

We start with considering three standard measures of nutritional status in early childhood. The first measure, birth weight, reflects prenatal nutritional intakes. The second and the third are respectively the weight-for-age and height-for-age z-scores at age 12 months, both of which incorporate both prenatal and postnatal nutritional developments. In contrast to some previous studies we do not find strong associations of birth weight with educational outcomes. Probably this is because in the 21 st century Vietnamese context, low birth weight is much less prevalent than in many of the data sets used for previous studies because of their use of twins or because they were for 20th century low- and middle-income contexts with higher prevalence of low birth weight. The estimates for birth weight in the current study are consistent with those in Gupta et al. (2013). In both studies birth weight predicts anthropometric, but not the other outcomes. Our results further are consistent with the studies related to the Dutch famine in 1944–1945. This famine led to lower birth weights of infants whose mothers experienced severe nutritional deprivation during their pregnancy. Stein et al. (1972), however, find that at the age of 19 years there were no detectable adverse effect on cognitive ability of the children born around the time and location of the event. Back to the current study, even for the outcomes for which birth weight has the most significant predictive power – anthropometries at age eight years – it predicts a smaller part of the variance than predicted by any of the weight or height z-scores.

Weight-for-age and height-for-age z-scores at age 12 months are strongly associated with all the anthropometric outcomes and educational outcomes at age eight years except the former does not predict the receptive vocabulary outcome. Together with the controls, height-for-age at age 12 months predicts half of the variation in height-for-age at age eight years (more than that predicted by weight-for-age at 12 months for weight-for-age at eight years of age). Correspondingly but not equivalently, weight-for-age at 12 months together with the controls predicts less than half of the variation in mid-childhood weight-for-age. That is evidence for some difference between height and weight in their predictive power – more evidence is summarized below.

Closely related to the aforementioned weight and height z-scores are their postnatal components. We define the concepts of unpredicted weight gain (height growth) in the first year of life as the parts of weight-for-age (height-for-age) z-scores that are uncorrelated with birth weight and all the control factors, including mother’s anthropometrics and household socioeconomic status. These unpredicted components of the weight and height z-scores are substantial. In fact, the standard deviations of the variables on birth weight, the unpredicted weight gain, and the unpredicted height growth are 0.43, 0.92 and 0.94 respectively. The sizes of anthropometric outcomes at age eight years associated with the standard deviation of unpredicted weight gain (height growth) are more than double those of a standard deviation in birth weight. Unlike birth weight, the unpredicted weight gain in the first year of life is strongly associated with two of the educational outcomes. Unpredicted height growth in the same period is strongly associated as well with the receptive vocabulary outcome at age eight years.

Our study has limitations, including inadequate information on birth outcomes other than birth weight and no information on anthropometrics at age 24 months, which often has been emphasized in the nutritional literature as particularly important (e.g., Victora et al., 2008, Victora et al., 2010). With regard to the latter, however Schott et al. (2013) report that “in analysis using the Institute for Nutrition in Central America and Panama (Guatemala) nutritional supplementation study data (Stein et al., 2008:286), in which there were multiple measures over the first 7 years of age, we found that HAZ measures at ages 6–17.9 months predict well HAZ at age 24 months (for ages 6, 12, and 18 months, correlations with HAZ at age 24 months were r = 0.74, 0.83, 0.91, respectively). If similar correlations across ages also hold for populations of Young Lives countries [in our case, particularly in Vietnam], the cross-sectional patterns in HAZ at ages 6–18 months that were observed in the Young Lives data represent fairly well cross-sectional patterns in HAZ that likely held for these same children at 24 months, even if the overall distribution in HAZ may have declined fairly substantially from 12 to 24 months.”

Despite these limitations, our investigation sheds considerable light on how, in the context of Vietnam in the early 21st century, early-life nutritional indicators, together with family characteristics, link to latter outcomes. We find the associations of the socioeconomic status with all the mid-childhood outcomes consistently strong. Our estimates imply a standard deviation higher unpredicted weight gain (or unpredicted height growth) in the first year of life has larger associations with height and weight outcomes at age eight years than a standard deviation higher birth weight. Parents might expect significant adverse outcomes in height and weight at age eight years for children born with lower birth weights. However, expectations about mid-childhood outcomes can change substantially at age one year thanks to developments that were unforeseen at birth because the unpredicted weight gain (height growth) over the first postnatal year are by construction independent of birth weight, socioeconomic status and maternal anthropometry. In addition to the aforementioned difference between the prenatal and postnatal nutrition with respect to their magnitudes of associations with the outcomes, we find difference between the postnatal indicators on height versus weight. That is, there is a component in height-for-age (at age one year) that adds explanatory power for all the outcomes beyond that due to the linear combination of birth weight, weight-for-age z-score (at age one year) and all the controls.

## Figures and Tables

**Table A1:** OLS Regression of birth weight (in grams).

	Coefficient	t-statistic
If male	63.57***	4.01
The baby was very large	752.75***	9.79
The baby was large	402.86***	17.35
The baby was small	−333.92***	−14.54
The baby was very small	−625.65***	−12.35
Number of weeks premature	−83.99***	−10.25
If the child is first	−93.97***	−5.86
Born in 2002	45.05**	2.13
Mother ethnic minority	−74.35*	−1.85
Mother height	8.54***	6.11
Mother BMI	10.50***	3.45
Mother completed LSE	41.19**	2.26
Wealth index Round 1	76.29	1.31
Urban	−42.57	−1.44
Mountainous	−133.33***	−3.94
Red River Delta	−112.68***	−4.19
Mekong Delta	−135.93***	−4.24

Number of observations	1673	
R-squared	0.49	

*Notes*: Dummies for two specific sentinel sites do not display;

* p< 0.1.

** p < 0.05.

*** p < 0.01.

**Table A2:** Estimates of coefficients of nutritional variables. Subsample of only the observations with non-missing data on birth weight

Model	Variables other than controls	WAZ8	HAZ8	EGRA	Math	PPVT
0	Norm. birth weight (y_0_)	0.157***	0.145***	0.005	0.022	−0.008
		(0.031)	(0.028)	(0.030)	(0.036)	(0.025)

1W	Weight-for-age at 1 year	0.566***	0.376***	0.118***	0.083***	0.022
		(0.022)	(0.021)	(0.023)	(0.023)	(0.024)

1H	Height-for-age at 1 year	0.464***	0.508***	0.112***	0.088***	0.049**
		(0.023)	(0.019)	(0.022)	(0.018)	(0.018)

2W	Unpredicted weight gain (y_1_)	0.510***	0.334***	0.110***	0.075***	0.022
		(0.020)	(0.019)	(0.021)	(0.020)	(0.021)

2H	Unpredicted height growth (y_2_)	0.443***	0.489***	0.112***	0.085***	0.051***
		(0.023)	(0.021)	(0.021)	(0.018)	(0.018)

3W	Birth weight (y_0_)	0.147***	0.138***	0.003	0.020	−0.008
		(0.024)	(0.023)	(0.030)	(0.035)	(0.025)
	Unpredicted weight gain (y_1_)	0.509***	0.332***	0.110***	0.075***	0.022
		(0.020)	(0.019)	(0.021)	(0.020)	(0.021)

3H	Birth weight (y_0_)	0.145***	0.132***	0.002	0.020	−0.009
		(0.026)	(0.022)	(0.029)	(0.035)	(0.025)
	Unpredicted height growth (y_2_)	0.441***	0.487***	0.112***	0.085***	0.051***
		(0.022)	(0.020)	(0.021)	(0.018)	(0.018)

4HW	Birth weight (y_0_)	0.145***	0.132***	0.002	0.020	−0.009
		(0.024)	(0.022)	(0.029)	(0.035)	(0.025)
	Unpredicted height growth (y_2_)	0.435***	0.487***	0.111***	0.085***	0.051***
		(0.020)	(0.020)	(0.021)	(0.019)	(0.018)
	Conditional unpredicted WAZ1 (y_3_)	0.291***	0.005	0.048**	0.024	−0.017
		(0.023)	(0.019)	(0.020)	(0.018)	(0.018)

4WH	Birth weight (y_0_)	0.145***	0.132***	0.002	0.020	−0.009
		(0.024)	(0.022)	(0.029)	(0.035)	(0.025)
	Unpredicted weight gain (y_1_)	0.511***	0.338***	0.111***	0.076***	0.023
		(0.019)	(0.016)	(0.021)	(0.020)	(0.021)
	Conditional unpredicted HAZ1 (y_4_)	0.116***	0.350***	0.047**	0.045***	0.049***
		(0.024)	(0.022)	(0.020)	(0.016)	(0.014)

*Notes*: The variables on early childhood nutrition and on test scores in EGRA, Math and PPVT are normalized by the sample standard deviations; Number of observations: 1556; the standard errors (in parentheses, underneath the coefficients) are robust to heteroscedasticity.

^*^p < 0.1.

** p < 0.05.

*** p < 0.01.

## Appendix A Method for imputation of missing birth weight

To deal with the issue on missing birth weight data, we use both the original data on birth weight and imputed birth weight for cases in which observed birth weight is not available, but only perceived relative size at birth. Mothers were asked, “Did you think the child was large, average or small at birth” and the codes are given 1–5 for the answers “Very large”, “Large”, “Average”, “Small”, and “Very small” correspondingly. We use the results of OLS regression to fill in the imputed birth weight for the children with missing birth weight. These imputed values are between the lower and the upper limits for actual birth weights. Among the age-targeted children, 232 imputed values are applied for the missing ones. The average for the imputed values are 3087 g, while that for the no missing cases is 3101 g. The results of the OLS regression are presented in the following Table A1.

## Footnotes

1 Both papers present Ordinary Least Squares associations.

2 Behrman and Rosenzweig (2004) and Rosenzweig and Zhang (2013) primarily use twins and present birth weight distributions for twins that are significantly to the left of those for singletons in the same populations. Most of the other studies mentioned above use both singletons and twins in proportions roughly representative of the populations studied.

3 There were 8 children older than 17.9 months and 13 children under 6 months in Round 1.

4 Of the children 6–17.9 months in Round 1, there are 32 cases with values in either height or weight that are different from the medians in the WHO’s reference population by more than five standard deviations. Additionally, we find 13 observations with change in either height or weight exceeding four standard deviation from one survey round to the next.

5 3.5 kg is about the mean plus one standard deviation for birth weight. We also explored including a dichotomous variable for low birth weight (<2.5 kg) but found that it had no predictive power beyond that of birth weight.

6 In September 2007, for instance, 95% of the children born in 2001 were in primary schools, and 5% were in preschools. In the same month, nearly 30% of those born in 2002 were in grade one.

7 For ages *i *= 6 and *i* = 17 months the averages are calculated for the children in two, rather than three, consecutive monthly age groups.

8 The Young Lives data do not have multiple measurements of HAZ for the same children at different ages in infancy. However the well-known Guatemalan Institute for Nutrition in Central America and Panama (INCAP) and Cebu Longitudinal Health and Nutrition Survey (CLHNS) longitudinal studies (Adair et al., 2010, Stein et al., 2008), both of which are members of the influential COHORTS group (Richter et al., 2012) and both of which have been used in influential studies about the longer-run effects of early-life undernutrition (Adair et al., 2009, Black et al., 2013, Fall et al., 2011, Kuzawa et al., 2012, Lundeen et al., 2014, Martorell et al., 2010, Stein et al., 2010, Victora et al., 2008), do have data on HAZ for the same children at ages 6, 12 and 18 months. For INCAP, the correlation between HAZ at 6 month and HAZ at 12 months is 0.84, and the correlation between HAZ at 12 months and HAZ at 18 months is 0.89. The corresponding figures for Cebu are 0.76 and 0.85. While the correlations between HAZ at 6 and 12 months and between HAZ at 12 and 18 months are not perfect, they are significant and fairly substantial. If similar correlations hold for the Young Lives data, our procedure yields good but not perfect estimates of HAZ at age 12 months even for the youngest and oldest children in the samples at Round 1 (and, of course, better estimates the closer the age at measurement was to age 12 months). If the measurement error due to this procedure is random, our coefficient estimates are likely to be biased towards zero.

9 The three dummy variables are: (i) floor equals one if the dwelling floor is made of a finished material (such as cement, tile or a laminated material); (ii) wall equals one if house has brick or plastered walls; and (iii) roof equals one if house has a sturdy roof (such as corrugated iron, tiles or concrete).

10 The basic consumer durables included are: radios, refrigerators, bicycles, motorcycles, cars, mobile phones, landline phones, fans, and televisions.

11 The list of assets in the Young Lives wealth index is similar to that in Filmer and Pritchett (2001), who use principal components to determine the weights for an index based on individual asset variables. As the coefficients are all positive in the Young Lives wealth index and in the index in Filmer and Pritchett (2001), these two indices are expected to be fairly correlated. Filmer and Scott (2012) report that such asset indices are robust to a range of definitions.

12 *Source*: http://web.cema.gov.vn/modules.php?name=Doc&op=detaildoc&pid=150.

13 A robustness check shows that if these two dummy variables are dropped, the statistical significance of estimates for the early childhood indicators are not changed substantially.

14 The assumption may seem too strong in the case of the wealth index. However, because the wealth index is included in Eq. (3), an inclusion/exclusion of this factor in any of Eqs. (1a), (1b), (2a) and (2b) does not lead to any change in the estimates (Eq. (3)) for the unpredicted variables. We include all the controls in all equations in order to isolate the effects of the unpredicted variables.

15 The dummy variables on birth weight >3.5 kg and on imputed birth weight are not included in Model C.

16 As for the current study, Krishna et al. (2016) work with Young Lives data. However, the only outcome measure considered in their study is height-for-age, and they do not investigate the impacts of unpredicted weight gain and height growth.
